# An unusual foreign object mimicking an odontoma in a patient with cleft alveolus: a case report

**DOI:** 10.1186/s13256-017-1433-x

**Published:** 2017-09-26

**Authors:** Nadeena Sri Swarnagupta Jayasuriya, Pallege Ralalage Chandana Lakmal Karunathilaka, Parakrama Wijekoon

**Affiliations:** 1 Department of Oral and Maxillofacial Surgery, Faculty of Dental Sciences, University of Peradeniya, Peradeniya, Sri Lanka; 2 Oral and Maxillofacial Surgery Unit, Dental Hospital (Teaching), Peradeniya, Sri Lanka

**Keywords:** Foreign body, Button battery, Cleft alveolus, Self-inflicted, Case report

## Abstract

**Background:**

The habit of inserting foreign objects into body cavities is seen in children and in adults with intellectual disability. Usually, the foreign objects cause chronic inflammation and local tissue destruction, which give rise to symptoms. Diagnosis at an asymptomatic stage is uncommon when the history is not suggestive. We describe a rare case where a foreign object was misdiagnosed as an odontoma in a patient with an alveolar cleft.

**Case presentation:**

A radiopaque round mass was noted on the radiograph of a 12-year-old Sinhalese boy who was awaiting an alveolar bone graft. Apart from problems related to the alveolar cleft and mild halitosis, he was otherwise healthy. This was suspected to be an odontoma in the cleft region. During alveolar bone graft surgery, a button battery was recovered that was later confirmed as having been self-inserted by the child. Alveolar bone graft surgery was delayed because of local chronic inflammation due to the foreign object. Three months later, complete healing of the site was noted when reexplored for alveolar bone grafting.

**Conclusions:**

It is important to include foreign objects in the radiological differential diagnosis in asymptomatic children. Furthermore, cone beam computed tomography should be considered in suspected cases. Early removal with thorough debridement causes minimal tissue destruction.

## Background

Foreign bodies cause serious harm due to chronic irritation and infection, in addition to the risk of aspiration by small children. Foreign objects (FOs) recovered from the head and neck region include fish bones, pieces of gauze, coins, buttons, beads, small pieces of toys, ectopic teeth, pieces of steel wire, nutshells, pieces of wood, artificial fingernails, and “opiomas” [1–5]. The most common site for impacted FOs is the nasal cavity. Other significant sites reported include the oral cavity (base of the tongue, hard palate), tonsils, piriform fossae, hypopharynx, cervical esophagus, and nasopharynx [1, 6].

Long-standing FOs in the head and neck are usually diagnosed after they give rise to symptoms [3, 4]. At the presymptomatic stage, an unsuspected FO is rarely confused with a normal structure or in the differential diagnosis involving radiological investigations. We report a case of an asymptomatic FO in an unrepaired alveolar cleft that was misdiagnosed as an odontoma on the basis of a plain radiograph.

## Case presentation

**Fig. 1:**
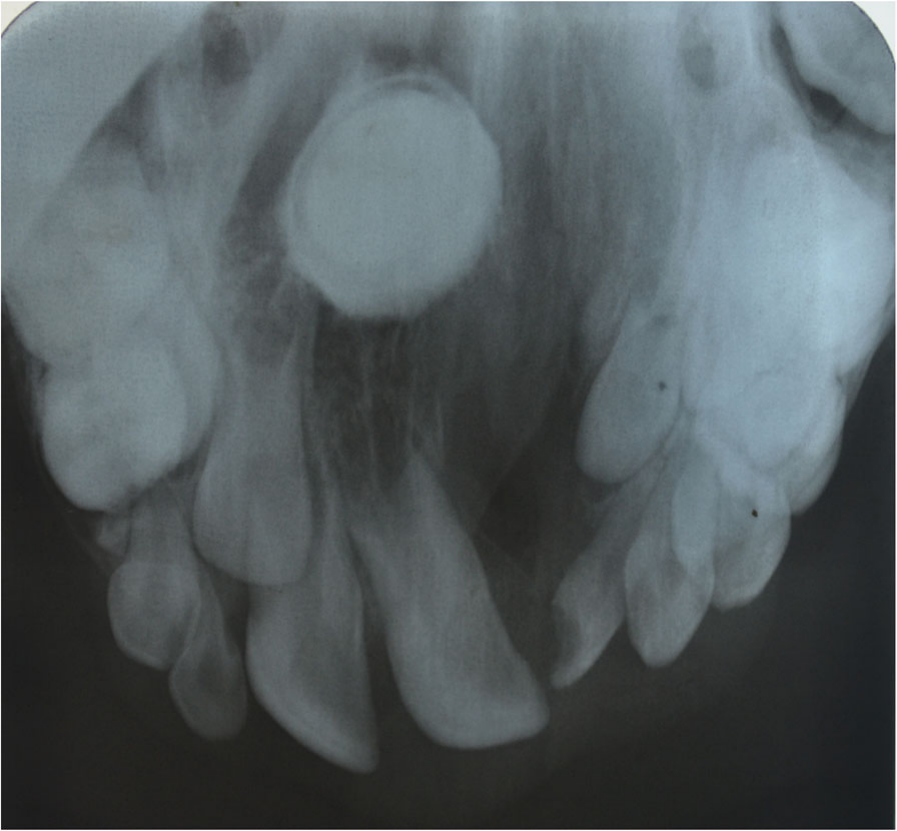
Upper standard occlusal radiograph showing a well-defined, circumscribed, radiopaque lesion in the midposterior palate. A rim of radiolucency mimicking a dental follicle was noted at the posterior margin

A 12-year-old Sinhalese boy from Kandy District, Sri Lanka, with a unilateral alveolar cleft was seen at a routine appointment in the cleft joint clinic of our institution. Apart from mild halitosis complained of by his mother, the boy was asymptomatic and was awaiting alveolar bone graft surgery. His halitosis was not clinically significant, and no accompanying discharges from his nostrils or oral cleft were noted. The palatal mucosa over the suspected lesion was noted to be slightly paler than the other regions. On palpation, the lesion was firm, nontender, and immobile. An upper standard occlusal radiograph through the alveolar cleft showed a well-demarcated, circumscribed, radiopaque mass in the palatal bone associated with the cleft (Fig. 1).

An odontoma or an ectopic tooth were the most likely diagnoses, considering the radiological appearance, which was of similar intensity to enamel; the appearance of a follicle-like structure; and the site of the lesion. To determine the exact location and the dimensions, cone beam computed tomography (CBCT) was suggested. The parents of the child did not consent to additional radiographic imaging, owing to fear of multiple radiation exposures and for financial reasons. Therefore, surgical removal with the patient under general anesthesia at the time of the alveolar bone graft surgery was planned.

**Fig. 2:**
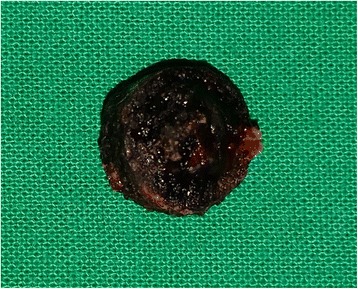
The lesion soon after extraction from the palate

**Fig. 3:**
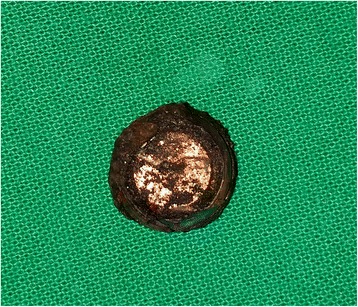
A cell-type button battery was seen after the lesion was cleaned

At the time of the surgery, a dark-colored, circumscribed lesion was noted to be impacted in the posterior part of the alveolar cleft. It was seen tightly fixed to the palatal mucosa due to fibrosis. After removal, cleaning, and examination of the lesion, a small button cell-type battery was identified (Figs. 2 and 3). A certain degree of fibrosis, bone resorption, and mucosal discontinuity was noted surrounding the FO; thus, alveolar bone graft surgery was considered unsuitable and postponed. Corrosion or leak of chemicals from the battery was not noted. Even after retrieval, the child denied inserting the object into his nose or mouth. Because he had no previous history, psychiatric referral was not considered.

## Discussion

The aerodigestive tract is a common site into which exogenous FOs are inserted. The child’s inquisitive nature and impulsive behavior may have contributed to such behavior, and due to fear, the child may have abstained from revealing this to adults [4].

The clinical presentation changed due to the object’s location, composition, and depth of penetration [5]. However, the encounter of an FO at an asymptomatic stage causes a dilemma in diagnosis, especially when previous history of FO insertion is absent.

FO impactions clinically misdiagnosed as palatal neoplasms and salivary gland tumors in the oral cavity are reported in the literature [4, 6]. To the best of our knowledge, we report the first case in which an FO mimicked an odontoma during radiographic evaluation.

The possible harmful effects of an impacted FO are aspiration, ingestion, local tissue irritation, and penetration into the body’s tissues. Subsequently, corrosive and toxic chemicals such as mercury, silver, alkaline manganese, and lithium of a cell-type battery can leak, causing lethal hazards to the patient [5]. The local tissue irritation may vary with the battery’s charge status [2]. Fortunately, in our patient, only mild tissue destruction due to chronic inflammation was noted. On subsequent reexploration for alveolar bone graft surgery, no significant tissue destruction was noted.

We feel that the conventional radiograph was a limitation leading to misdiagnosis. Due to finances involved, CBCT is not performed as a first-line radiological investigation in most cases in our practice.

## Conclusions

FO impaction in the head and neck area can happen among children. It is important to include FO impaction in the differential diagnosis because it can mimic different entities/pathologies in radiographic evaluation. In addition, advance imaging such as CBCT in suspected cases should be performed for further clarification.

## Abbreviations

CBCT: Cone beam computed tomography
FO: Foreign object
